# Prediction of postoperative infection in elderly using deep learning-based analysis: an observational cohort study

**DOI:** 10.1007/s40520-022-02325-3

**Published:** 2023-01-04

**Authors:** Pinhao Li, Yan Wang, Hui Li, Baoli Cheng, Shuijing Wu, Hui Ye, Daqing Ma, Xiangming Fang, the International Surgical Outcomes Study (ISOS) group in China, Ying Cao, Hong Gao, Tingju Hu, Jie Lv, Jian Yang, Yang Yang, Yi Zhong, Jing Zhou, Xiaohua Zou, Miao He, Xiaoying Li, Dihuan Luo, Haiying Wang, Tian Yu, Liyong Chen, Lijun Wang, Yunfei Cai, Zhongming Cao, Yanling Li, Jiaxin Lian, Haiyun Sun, Sheng Wang, Zhipeng Wang, Kenru Wang, Yi Zhu, Xindan Du, Hao Fan, Yunbin Fu, Lixia Huang, Yanming Huang, Haifang Hwan, Hong Luo, Pi-Sheng Qu, Fan Tao, Zhen Wang, Guoxiang Wang, Shun Wang, Yan Zhang, Xiaolin Zhang, Chao Chen, Weixing Wang, Zhengyuan Liu, Lihua Fan, Jing Tang, Yijun Chen, Yongjie Chen, Yangyang Han, Changshun Huang, Guojin Liang, Jing Shen, Jun Wang, Qiuhong Yang, Jungang Zhen, Haidong Zhou, Junping Chen, Zhang Chen, Xiaoyu Li, Bo Meng, Haiwang Ye, Xiaoyan Zhang, Yanbing Bi, Jianqiao Cao, Fengying Guo, Hong Lin, Yang Liu, Meng Lv, Pengcai Shi, Xiumei Song, Chuanyu Sun, Yongtao Sun, Yuelan Wang, Shenhui Wang, Min Zhang, Rong Chen, Jiabao Hou, Yan Leng, Qing-tao Meng, Li Qian, Zi-ying Shen, Zhong-yuan Xia, Rui Xue, Yuan Zhang, Bo Zhao, Xian-jin Zhou, Qiang Chen, Huinan Guo, Yongqing Guo, Yuehong Qi, Zhi Wang, Jianfeng Wei, Weiwei Zhang, Lina Zheng, Qi Bao, Yaqiu Chen, Yijiao Chen, Yue Fei, Nianqiang Hu, Xuming Hu, Min Lei, Xiaoqin Li, Xiaocui Lv, Jie Lv, Fangfang Miao, Lingling Ouyang, Lu Qian, Conyu Shen, Yu Sun, Yuting Wang, Dong Wang, Chao Wu, Liyuan Xu, Jiaqi Yuan, Lina Zhang, Huan Zhang, Yapping Zhang, Jinning Zhao, Chong Zhao, Lei Zhao, Tianzhao Zheng, Dachun Zhou, Haiyan Zhou, Ce Zhou, Kaizhi Lu, Ting Zhao, Changlin He, Hong Chen, Shasha Chen, Jie He, Lin Jin, Caixia Li, Yuanming Pan, Yugang Shi, Xiao Hong Wen, Guohao Xie, Kai Zhang, Bing Zhao, Xianfu Lu, Feifei Chen, Qisheng Liang, Xuewu Lin, Yunzhi Ling, Gang Liu, Jing Tao, Lu Yang, Jialong Zhou, Fumei Chen, Zhonggui Cheng, Hanying Dai, Yunlin Feng, Benchao Hou, Haixia Gong, Chun hua Hu, Haijin Huang, Jian Huang, Zhangjie Jiang, Mengyuan Li, Jiamei Lin, Mei Liu, Weicheng Liu, Zhen Liu, Zhiyi Liu, Foquan Luo, Longxian Ma, Jia Min, Xiaoyun Shi, Zhiping Song, Xianwen Wan, Yingfen Xiong, Lin Xu, Shuangjia Yang, Qin Zhang, Hongyan Zhang, Huaigen Zhang, Xuekang Zhang, Lili Zhao, Weihong Zhao, Weilu Zhao, Xiaoping Zhu, Yun Bai, Linbi Chen, Sijia Chen, Qinxue Dai, Wujun Geng, Kunyuan Han, Xin He, Luping Huang, Binbin Ji, Danyun Jia, Shenhui Jin, Qianjun Li, Dongdong Liang, Shan Luo, Lulu Lwang, Yunchang Mo, Yuanyuan Pan, Xinyu Qi, Meizi Qian, Jinling Qin, Yelong Ren, Yiyi Shi, Junlu Wang, Junkai Wang, Leilei Wang, Junjie Xie, Yixiu Yan, Yurui Yao, Mingxiao Zhang, Jiashi Zhao, Xiuxiu Zhuang, Yanqiu Ai, Du Fang, Long He, Ledan Huang, Zhisong Li, Huijuan Li, Yetong Li, Liwei Li, Su Meng, Yazhuo Yuan, Enman Zhang, Jie Zhang, Shuna Zhao, Zhenrong Ji, Ling Pei, Li Wang, Chen Chen, Beibei Dong, Jing Li, Ziqiang Miao, Hongying Mu, Chao Qin, Lin Su, Zhiting Wen, Keliang Xie, Yonghao Yu, Fang Yuan, Xianwen Hu, Ye Zhang, Wangpin Xiao, Zhipeng Zhu, Qingqing Dai, Kaiwen Fu, Rong Hu, Xiaolan Hu, Song Huang, Yaqi Li, Yingping Liang, Shuchun Yu, Zheng Guo, Yan Jing, Na Tang, Wu Jie, Dajiang Yuan, Ruilin Zhang, Xiaoying Zhao, Yuhong Li, Hui-Ping Bai, Chun-Xiao Liu, Fei-Fei Liu, Wei Ren, Xiu-Li Wang, Guan-Jie Xu, Na Hu, Bo Li, Yangwen Ou, Yongzhong Tang, Shanglong Yao, Shihai Zhang, Cui-Cui Kong, Bei Liu, Tianlong Wang, Wei Xiao, Bo Lu, Yanfei Xia, Jiali Zhou, Fang Cai, Pushan Chen, Shuangfei Hu, Hongfa Wang, Wu Jie, Qiong Xu, Liu Hu, Liang Jing, Jing Li, Bin Li, Qiang Liu, Yuejiang Liu, Xinjian Lu, Zhen Dan Peng, Xiaodong Qiu, Quan Ren, Youliang Tong, Zhen Wang, Jin Wang, Yazhou Wen, Qiong Wu, Jiangyan Xia, Jue Xie, Xiapei Xiong, Shixia Xu, Tianqin Yang, Ning Yin, Jing Yuan, Qiuting Zeng, Baoling Zhang, Kang Zheng, Jing Cang, Shiyu Chen, Du Fang, Yu Fan, Shuying Fu, Xiaodong Ge, Baolei Guo, Wenhui Huang, Linghui Jiang, Xinmei Jiang, Lin Jin, Yi Liu, Yan Pan, Yun Ren, Qi Shan, Jiaxing Wang, Fei Wang, Chi Wu, Xiaoguang Zhan

**Affiliations:** 1 Department of Anesthesiology, The First Affiliated Hospital, Zhejiang University School of Medicine, 79 Qingchun Road, Hangzhou, China; 2 Division of Anaesthetics, Pain Medicine and Intensive Care, Department of Surgery and Cancer, Faculty of Medicine, Imperial College London, Chelsea and Westminster Hospital, London, UK

**Keywords:** Artificial intelligence, Deep learning, Elderly patients, Machine learning, Postoperative infections

## Abstract

Elderly patients are susceptible to postoperative infections with increased mortality. Analyzing with a deep learning model, the perioperative factors that could predict and/or contribute to postoperative infections may improve the outcome in elderly. This was an observational cohort study with 2014 elderly patients who had elective surgery from 28 hospitals in China from April to June 2014. We aimed to develop and validate deep learning-based predictive models for postoperative infections in the elderly. 1510 patients were randomly assigned to be training dataset for establishing deep learning-based models, and 504 patients were used to validate the effectiveness of these models. The conventional model predicted postoperative infections was 0.728 (95% CI 0.688–0.768) with the sensitivity of 66.2% (95% CI 58.2–73.6) and specificity of 66.8% (95% CI 64.6–68.9). The deep learning model including risk factors relevant to baseline clinical characteristics predicted postoperative infections was 0.641 (95% CI 0.545–0.737), and sensitivity and specificity were 34.2% (95% CI 19.6–51.4) and 88.8% (95% CI 85.6–91.6), respectively. Including risk factors relevant to baseline variables and surgery, the deep learning model predicted postoperative infections was 0.763 (95% CI 0.681–0.844) with the sensitivity of 63.2% (95% CI 46–78.2) and specificity of 80.5% (95% CI 76.6–84). Our feasibility study indicated that a deep learning model including risk factors for the prediction of postoperative infections can be achieved in elderly. Further study is needed to assess whether this model can be used to guide clinical practice to improve surgical outcomes in elderly.

**Supplementary Information:**

The online version contains supplementary material available at 10.1007/s40520-022-02325-3.

## Introduction

Infection is the leading cause of morbidity and mortality in hospitalized patients [1–4]. Owing to the aged deterioration of the whole body system including compromised immune function, elderly patients are susceptible to infection after surgery [5, 6]. Given the aging society globally, more and more elderly patients receive surgery. However, those patients face an increased risk of postoperative infections. Unfortunately, the risk prediction of postoperative infections in elderly is largely lacking [7, 8].

Artificial intelligence is emerging to be used for addressing medical challenges, for example, sepsis [9–11]. Recent advance in deep learning, one of the types of artificial intelligence, has been shown to help the learning feature of data representations and improve modeling performance in different settings [12–15]. For example, several studies demonstrated that a deep learning-based approach has achieved great success in predicting events in clinical practice [16, 17]. However, the use of deep learning to detect postoperative infections in elderly patients is limited. The current study used a deep learning-based strategy to predict postoperative infections in elderly patients following surgery. The primary objective of this study was to develop and validate deep learning models for predicting postoperative infections in elderly patients which focused on in-hospital assessment for infections. In addition, we sought to examine whether the deep learning neural network model is superior to the conventional regression model in predicting the risk of developing postoperative infections using the area under the receiver operating characteristic curve (AUC) to calculate sensitivity and specificity.

## Methods

### Study design and population

This study was part of the International Surgical Outcomes Study (ISOS) project, an international observational cohort study of complications following elective surgery. ISOS was registered prospectively with an international trial registry (ISRCTN51817007) [18]. The study was approved by Research Ethics Committee, The First Affiliated Hospital, Zhejiang University School of Medicine (reference: 2014-011), and all methods were performed in accordance with the relevant guidelines. The current dataset with permission to be reported was from elderly patients (≥ 60 years) who had elective surgery and at least one-night hospital stay and were recruited from 28 hospitals (Online Appendix 1) in China between April and June 2014. Patients with emergency surgery, day-case surgery, or interventional radiotherapy were excluded. Patients’ baseline characteristics included gender, current smoker, ASA score, comorbidities (coronary artery disease, heart failure, diabetes mellitus, metastatic cancer, cirrhosis, stroke, COPD/asthma, other), and blood measurements (hemoglobin, serum creatinine, sodium, and leucocytes) were collected. Surgery-related data included surgical procedures (orthopedic, gynecology, urology and kidney, upper gastrointestinal, lower gastrointestinal, hepatobiliary, vascular, breast, head and neck, plastics and cutaneous, cardiac, thoracic, and other), anesthetic technique (general, spinal, epidural, and sedation/local), laparoscopic surgery, cancer surgery, and the severity of surgery (minor, intermediate, and major), and surgical checklist was harvested. Postoperative infections included urinary tract infection, bloodstream infection, superficial surgical site infection, deep surgical site infection, body cavity infection, and pneumonia. Infections were assessed according to the United States of America Centers for Disease Control (CDC) definitions of infections [19]. The detailed definitions of each infection are presented in Online Appendix 2 [20]. The diagnosis of postoperative infections was conducted by one study team member and verified by a second team member. The diagnostic accordance rate of postoperative infections was 95% by two study team members. Patients’ informed consent was exempted as all data were anonymized and were already recorded as part of routine clinical care.

**Fig. 1:**
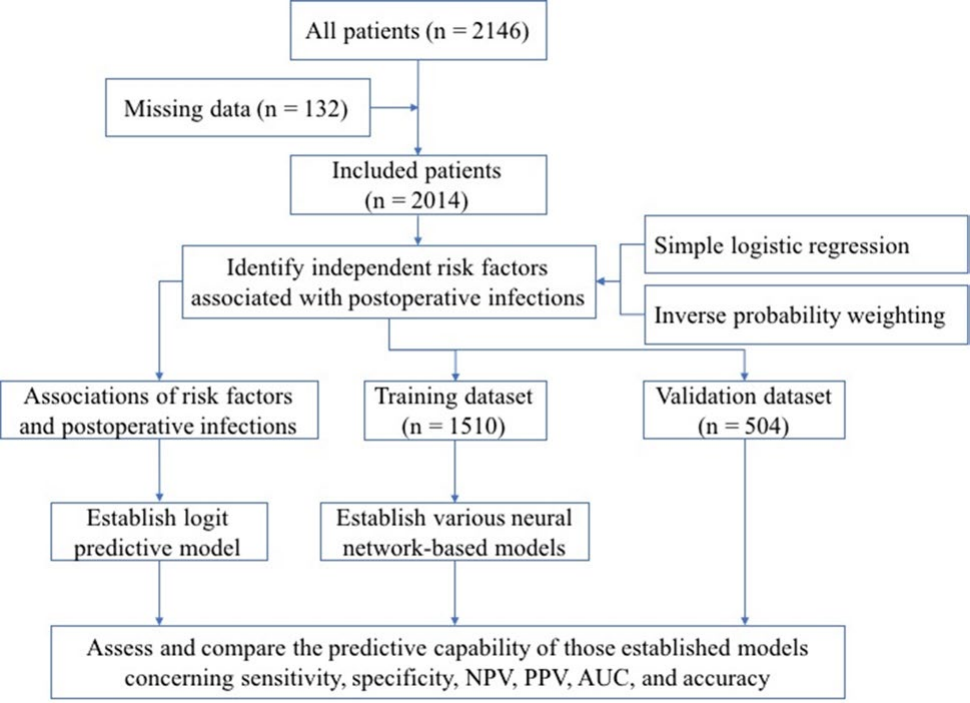
Flow diagram of patients through the study. *NPV* negative predictive value, *PPV* positive predictive value, *AUC* area under the receiver operating characteristic curve (ROC)

A total of 2014 elderly patients were recruited (Fig. 1) to identify independent risk factors for postoperative infections by using the inverse probability (IP) weighting method. The associations derived from IP weighting of those risk factors and postoperative infections were used to construct the conventional logistic regression predictive model. We assessed the predictive capability of the established conventional model for infections using sensitivity, specificity, negative predictive value (NPV), positive predictive value (PPV), and AUC. In addition, we split the original dataset into training and validation datasets with a 3:1 ratio to develop and validate the deep learning model. Of those, 1510 patients were randomly assigned for the training dataset and establishing various neural-network-based predictive models for postoperative infections. The remaining 504 patients were used to assess the sensitivity, specificity, NPV, PPV, and accuracy of various neural network-based models. We assigned more patients to the training dataset to ensure a well-trained neural network.

### Neural network analysis

In this study, deep learning is one of the machine learning models that use multilayered neural networks whose hierarchical computational design is partly inspired by a biological neuronal structure and was used to generate the output of the probability of infection complications after surgery [21]. The output layer consists of the response variables (Supplemental Fig. 1). For each neuron, a weight is attached, indicating the corresponding neuron’s effect and all data past the neural network signals. The signals are processed first by integrating all incoming signals and activation functions that transform the neuron's output. For a specific neural network, the observed data are used to train the neural network, in which the neural learns the approximation of the relationship by iteratively adapting its parameters.

We fitted deep learning models using the training dataset and evaluated prediction performances using the validation set. Hyperparameter (e.g., weights) tuning to identify the optimal values for parameters that were learned during the training process was performed using a back-propagation algorithm iteratively based on the training dataset, referred to as candidate neural network predictive models. Then, we selected the optimal neural network predictive model in terms of the maximum AUC value among those candidate neural networks based on the validation dataset. We selected the optimal threshold with Youden’s index (i.e., sensitivity + specificity—1) based on the validation dataset in terms of a range of candidate thresholds. Based on the optimal threshold, we further calculated the corresponding sensitivity, specificity, NPV, and PPV. Finally, the model comparison was carried out using AUC for classification accuracy of the probability of patients with postoperative infections, in which the higher values, the better accuracy of the predictive model.

### Statistical analysis

Data were presented as patient’s number (percentage) or odds ratio (OR) and 95% confidence interval (95% CI) where appropriate. All analyses were conducted using R software 3.6.2, with a neuralnet package for training and establishing the neural network predictive models and a pROC package to calculate the sensitivity, specificity, NPV, PPV, AUC, and accuracy. Confidence intervals were calculated based on the bootstrap method with 2000 replicates. A statistical significance was set at a level of *P* < 0.05.

## Results

### Independent risk factors associated with postoperative infections in elderly patients

**Fig. 2:**
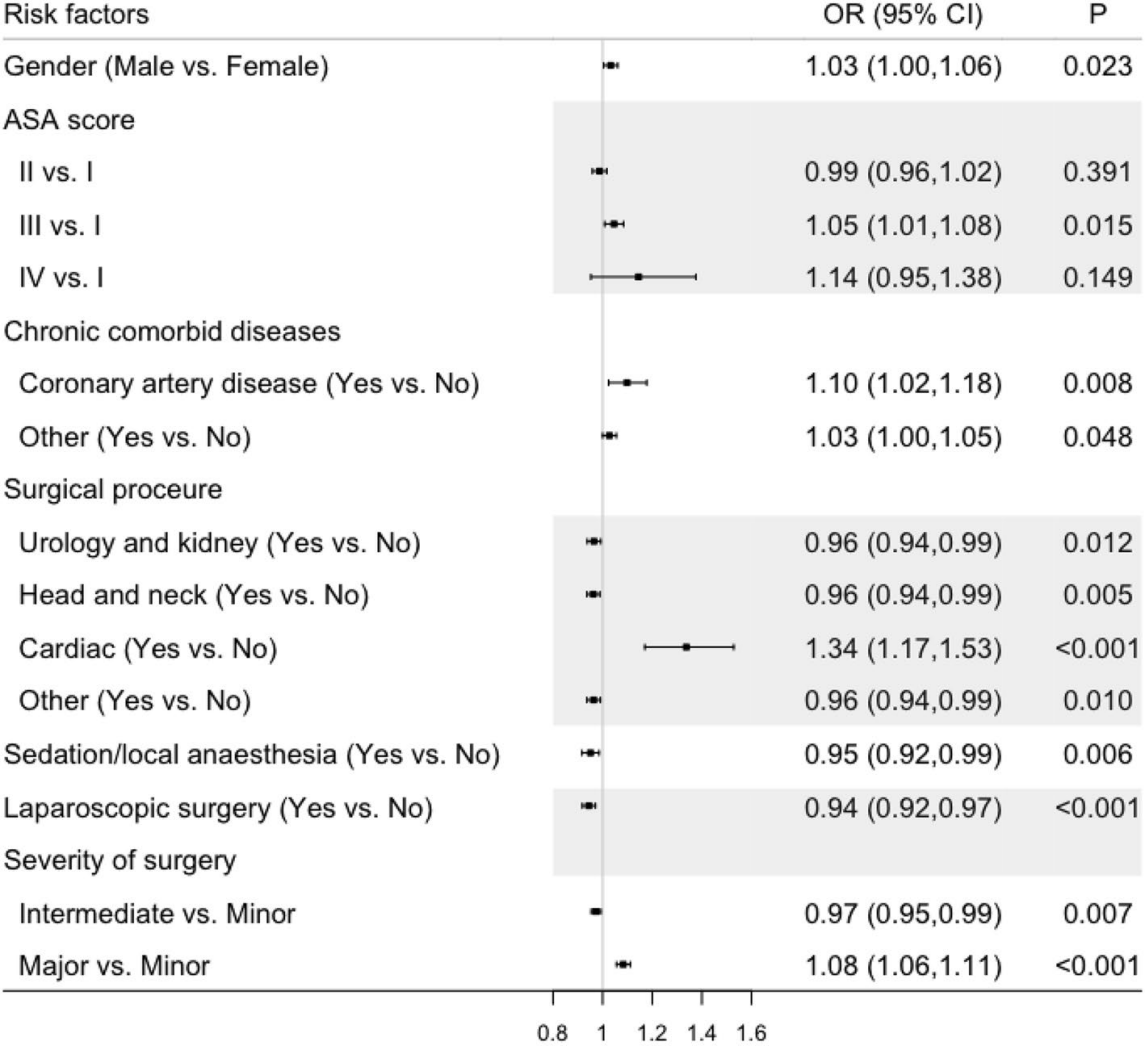
Adjusted odds ratio and 95% confidence interval for independent risk factors using the inverse probability weighting method. *ASA* American Society of Anesthesiology

Of 2014 patients, there were 12 potential risk factors associated with postoperative infections using simple logistic regression (Supplemental Table 1). 11 risk factors were associated with postoperative infections after multivariable adjustment as summarized in Table 1. Male patients had an odds ratio (OR) for postoperative infections of 1.03 (95% CI 1.00–1.06) compared with female patients. Using the lowest ASA score as the reference, ASA III was independently associated with an increased risk of postoperative infections (OR 1.05; 95% CI 1.01–1.08; *P* = 0.015). Coronary artery disease had an OR for postoperative infections of 1.10 (95% CI 1.02–1.18; *P* = 0.008). Sedation/local anesthesia was independently associated with a reduced risk of postoperative infections with an OR of 0.95 (95% CI 0.92–0.99; *P* = 0.006). Laparoscopic surgery was independently associated with a decreased risk of postoperative infections (OR 0.94; 95% CI 0.92–0.97; *P* < 0.001). In surgical procedures, independent risk factors of postoperative infections included urology and kidney surgery (OR 0.96; 95% CI 0.94–0.99), head and neck surgery (OR 0.96; 95% CI 0.94–0.99), and cardiac surgery (OR 1.34; 95% CI 1.17–1.53). Major surgery was independently associated with an increased risk of postoperative infections with an OR of 1.08 (95% CI 1.06–1.11; *P* < 0.001), but intermediate surgery was independently associated with a decreased risk of postoperative infections with an OR of 0.97 (95% CI 0.95–0.99; *P* = 0.007). The forest plot for independent risk factors is shown in Fig. 2. In addition, more clinical aspects and information related to infections are presented in Supplemental Tables 2 and 3.

**Table 1:** Independent risk factors for patients with postoperative infections

Independent risk factors	Adjusted	
	OR (95%CI)	*P*
Gender (male vs. female)	1.03 (1.00, 1.06)	0.023
ASA score		
I	Reference	
II	0.99 (0.96, 1.02)	0.391
III	1.05 (1.01, 1.08)	0.015
IV	1.14 (0.95, 1.38)	0.149
Chronic comorbid disease (yes vs. no)		
Coronary artery disease	1.10 (1.02, 1.18)	0.008
Other	1.03 (1.00, 1.05)	0.048
Surgical procedure (yes vs. no)		
Urology and kidney	0.96 (0.94, 0.99)	0.012
Head and neck	0.96 (0.94, 0.99)	0.005
Cardiac	1.34 (1.17, 1.53)	< 0.001
Other	0.96 (0.94, 0.99)	0.010
Sedation/local anesthesia	0.95 (0.92,0.99)	0.006
Laparoscopic surgery	0.94 (0.92, 0.97)	< 0.001
Severity of surgery		
Minor	Reference	
Intermediate	0.97 (0.95, 0.99)	0.007
Major	1.08 (1.06, 1.11)	< 0.001

*ASA* American Society of Anesthesiologists

### The conventional logit predictive model based on associations obtained from the IP weighting method

**Fig. 3:**
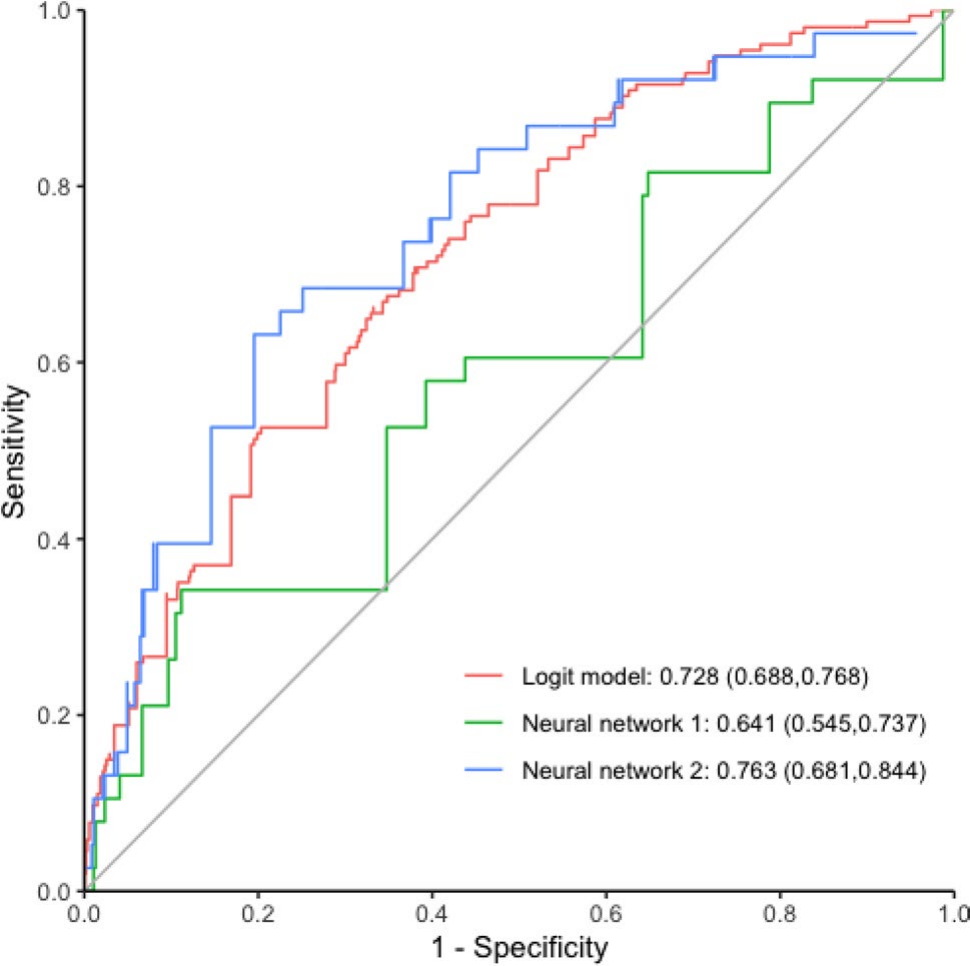
Comparison of predictive sensitivity and specificity of postoperative infections with the conventional model, neural network model I, and neural network model II assessed under the receiver operating characteristic curve (ROC)

To investigate the conventional logit predictive model, we calculated the coefficients using associations of independent risk factors with postoperative infections (Supplemental Table 4). The final logit predictive model is shown in Online Appendix 3. The receiver operating characteristic (ROC) curve of the conventional model is shown in Fig. 3. The logit predictive model had an AUC for the prediction of postoperative infections of 0.728 (95% CI 0.688–0.768), a sensitivity of 66.2% (95% CI 58.2–73.6), and a specificity of 66.8% (95% CI 64.6–68.9) (Table 2). Furthermore, the accuracy of the logit predictive model for the prediction of postoperative infections was 0.667 (95% CI 0.646–0.688).

**Table 2:** Summary of the predictive performance of these three established models

Method	Cutoff	Sensitivity	Specificity	NPV	PPV	AUC
Logit model	0.510	0.662 (0.582, 0.736)	0.668 (0.646, 0.689)	0.960 (0.948, 0.970)	0.142 (0.117, 0.169)	0.728 (0.688, 0.768)
Neural network: 11-3-1	0.077	0.342 (0.196, 0.514)	0.888 (0.856, 0.916)	0.943 (0.917, 0.963)	0.200 (0.111, 0.318)	0.641 (0.545, 0.737)
Neural network: 28-24-1	0.077	0.632 (0.460, 0.782)	0.805 (0.766, 0.840)	0.964 (0.940, 0.980)	0.209 (0.139, 0.294)	0.763 (0.681, 0.844)

*NPV* negative predictive value, *PPV* positive predictive value, *AUC* area under receivers operating characteristic curve

### Development and validation of deep learning neural network model

The distributions of independent risk factors among training dataset according to the status of postoperative infections are presented in Supplemental Table 5. There were unbalanced distributions of risk factors including gender, ASA, coronary artery disease, head and neck surgery, cardiac surgery, sedation/local anesthesia, laparoscopic surgery, and severity of surgery between patients with and without postoperative infections in the training dataset. In addition, in the validation dataset, there were unbalanced distributions of risk factors including ASA, coronary artery disease, cardiac surgery, and severity of surgery between patients with and without postoperative infections (Supplemental Table 6).

All identified independent risk factors in the previous section were divided into two categories: risk factors relevant to baseline variables and risk factors relevant to the surgery. Based on these, deep learning predictive model I included risk factors related to the baseline variables, and deep learning predictive model II included risk factors associated with the baseline variables and those relevant to the surgery. Considering there is no fixed rule for deciding how many hidden layers and neurons should be used in neural network predictive models, we explored the number of neurons, ranging from one to two times of the inputs under a five layers model. We limited the third hidden layer only to include one neutron to facilitate the pooling of information.

Of those, there were a total of 132 neural networks using risk factors relevant to baseline variables. The AUC for possible structures of neural network predictive model I is shown in Supplemental Fig. 2. We found the optimal neural network predictive model with the best performance among those 132 possible neural networks with five layers with 6-11-3-1-1, including 1 input layer with 6 nodes of gender, ASA score (II), ASA score (III), ASA (IV), coronary artery disease and other diseases, 3 hidden layers, and 1 output layer (Supplemental Table 7). Supplemental Figure 3 shows the optimal structure of deep learning predictive model I. As shown in Fig. 3, the AUC measure of the ROC curve obtained by deep learning model I was 0.641 (95% CI 0.545–0.737). This predictive model had an overall sensitivity and specificity of 34.2% (95% CI 19.6–51.4) and 88.8% (95% CI 85.6–91.6), respectively, for the prediction of postoperative infections (Table 2). The accuracy of the deep learning model I for the prediction of postoperative infections was 0.847 (95% CI 0.813–0.878).

There were 756 neural networks related to all independent risk factors. The AUC for possible structures of neural network predictive model II is shown in Supplemental Fig. 4. We found the optimal neural network predictive model with the best performance among those 765 possible neural networks with five layers with 14-28-24-1-1, including 1 input layer with 14 nodes of gender, ASA score (II), ASA score (III), ASA score (IV), coronary artery disease, other diseases, urology, and kidney surgery, head and neck surgery, cardiac surgery, other surgery, sedation/local anesthesia, laparoscopic surgery, surgical severity (intermediate) and surgical severity (major), 3 hidden layers, and 1 output layer. (Supplemental Table 7). Supplemental Figure 5 shows the optimal structure of deep learning predictive model II. As shown in Fig. 3, the AUC measure of the ROC curve obtained by deep learning model II was 0.763 (95% CI 0.681–0.844). This predictive model had an overall sensitivity and specificity of 63.2% (95% CI 46–78.2) and 80.5% (95% CI 76.6–84), respectively, for the prediction of postoperative infections (Table 2). The accuracy of deep learning model II for the prediction of postoperative infections was 0.792 (95% CI 0.754–0.826).

## Discussion

In this observational cohort study, we applied a deep learning framework for the prediction of infections in elderly patients after surgery. We demonstrated that an artificial-intelligence model using deep learning neural networks can achieve a promising prediction of postoperative infections in elderly patients. Predictive performance was improved further when the deep learning-based model was derived with risk factors relevant to baseline clinical characteristics/measurements and surgery in combination. Our work indicates that using deep learning may guide clinical practice to prevent infections following surgery in elderly although further work is needed to validate machine learning for it to be a potential and ubiquitous integral part of routine clinical use for elderly surgical patients.

Deep learning is the process of training a neural network (a large mathematical function with millions of parameters) to perform a given task [14]. Many hidden layers neurons were used to produce increasing abstracted, nonlinear representations of the underlying data [22]. Considering the possible non-linear associations, an artificial neural network based on multiple-layer perceptions reflects a complex functional relationship between risk factors and responding variables with the back-propagation algorithm, the logit activation function, and error function. Deep learning uses back-propagation to indicate how a machine should change its internal parameters to predict the best desired output of responding variables [14]. This makes artificial neural networks to be a valuable toolbox for prediction. Indeed, deep learning models have been successfully applied in health care to predict clinical events, disease classification, and electronic health record data augmentation [23–27]. However, the use of deep learning to detect disease and complications in elderly patients is limited. In our study, we applied a deep learning approach to evaluate postoperative infections among elderly patients. Using a database of more than 2000 patients for training and validation, we found that the deep learning model had a high AUC of 0.763 for predicting postoperative infections among elderly patients after elective surgery. A fundamental finding in our study is that the deep learning model in predicting postoperative infections was better when compared with the standard regression model; the latter is often used as a traditional statistic method in building a prediction model. Recent studies have also demonstrated promising performance for predicting disease development using deep learning [28–30]. For example, a study reported a sensitivity of 96.8% at a specificity of 59.4% for detecting referable diabetic retinopathy [31]. In conjunction with these studies, our results further demonstrated that the deep learning algorithm can provide informative measures for the prediction of postoperative infections in elderly patients. The modeling approach reported here offers straightforward and computationally rapid guidance for clinicians to predict the likelihood of infections after surgery. We envision that our deep learning model can be used to identify high-risk elderly patients for postoperative infections. The findings may suggest the potential usage of our model to help doctors to justify interventions that may have a significant impact on perioperative management for elderly patients per se.

As elderly patients are the most frequent users of operative resources and are also the most vulnerable to postoperative infections, it is important for clinicians to gauge risk factors preoperatively [32, 33]. In this study, we identified 11 independent risk factors associated with postoperative infections including gender, ASA score, chronic comorbid diseases, surgical procedures, sedation/local anesthesia, laparoscopic surgery, and severity of surgery. More importantly, among the above risk factors, sedation/local anesthesia and laparoscopic surgery could reduce postoperative infections. These findings provide important evidence to clinical perioperative management for elderly patients. Interventions should be considered to tackle those risk factors to optimize the patient’s conditions before surgery. Indeed, some of these factors are modifiable by surgeons and anesthesiologists before surgery except gender, ASA score, and chronic comorbid disease. For example, sedation/local anesthesia was independently associated with a decreased risk of postoperative infections with an OR of 0.95 (95% CI 0.92–0.99; *P* = 0.006), suggesting anesthesiologists to select sedation or local anesthesia rather than general anesthesia when sedation or local anesthesia can meet the requirements of the surgery. In addition, laparoscopic surgery was also found to be associated with reduced postoperative infections with an OR of 0.94 (95% CI 0.92–0.97; *P* < 0.001), suggesting when feasible, laparoscopic rather than laparotomy surgery should be chosen. Therefore, some factors that reduce the risk of infections highlighted in our study could be preoperatively modified to improve the clinical outcome in elderly patients.

The strength of this study was that it collected a relatively large group of elderly patients undergoing elective surgery in multiple institutions. In addition, the baseline parameters and surgery-relevant characteristics applied in the deep learning model dovetail with a customary clinical workflow that can be routinely collected. However, this study is not without limitations. Clinical uses of AI have aroused skepticism including the difficulty of explaining the complex computational steps leading to a machine-generated clinical determination [34]. Although the deep learning model performs better than the conventional regression model in estimating the risk of postoperative infections in elderly patients, further studies are needed for validation. First, the deep learning model was built based on elderly patients admitted in the year of 2014, and further research is needed to verify the predictive performance of this model nowadays. Second, only data of routine clinical variables were used in the present study and we expect that the predictive performance could be boosted if individual assessment including the perioperative neurological status of elderly patients who often have delirium and/or cognitive impairment before and after surgery is incorporated into the model. Third, the present study focused on the prediction of infections including urinary tract infection, bloodstream infection, superficial and deep surgical site infection, body cavity infection, and pneumonia after elective surgery, which may not be well equipped for the specific types of infection. Fourth, considering the sensitivity and the specificity for predicting postoperative infections, our model seems to be relatively specific but not very sensitive. Further, study is needed to verify these findings and perhaps these can be improved by increasing the training data per se.

## Conclusions

We found that an artificial intelligence with a deep learning model has considerable advantages on predicting postoperative infections in elderly patients, indicating that the deep learning features are more discriminative and may have a better potential for predicting postoperative infections in elderly patients. Further investigation is warranted to improve the performance of the model and to understand how the model predicts postoperative infections even better.

## Supplementary Information

Below is the link to the electronic supplementary material.

Supplementary file1 (DOCX 788 kb)

## Data Availability

All data generated during the current study have been included in this published article and its supplementary file and are available from the corresponding author on reasonable request by email: xmfang@zju.edu.cn.
