# Supplementary material for: Prediction of postoperative infection in elderly using deep learning-based analysis: an observational cohort study
Source: Aging Clin Exp Res. 2023 Jan 4;35(3):639–47. doi: 10.1007/s40520-022-02325-3 (PMC10014765; doi:10.1007/s40520-022-02325-3)
Supplement: Supplementary file 1 — Supplementary file1 (DOCX 788 kb) [file 40520_2022_2325_MOESM1_ESM.docx]

Prediction of postoperative infection in elderly using deep learning-based analysis: An observational cohort study

Pinhao Li^#1^ PhD, Yan Wang^#1^ MD , Hui Li^1^ MD, Baoli Cheng^1^ PhD, Shuijing Wu^1^ MD, Hui Ye^1^ PhD, Daqing Ma^2^ MD, Xiangming Fang*^1^ MD, On behalf the International Surgical Outcomes Study (ISOS) group in China^†^

#These authors contributed equally to the work.

^1^Department of Anesthesiology, The First Affiliated Hospital, Zhejiang University school of medicine, Hangzhou, China

^2^Division of Anaesthetics, Pain Medicine and Intensive Care, Department of Surgery and Cancer, Faculty of Medicine, Imperial College London, Chelsea & Westminster Hospital, London, UK

†Members of the study group are listed in Appendix 1.

**Correspondence to:** Xiangming Fang, Department of Anesthesiology, The First Affiliated Hospital, Zhejiang University school of medicine, 79 Qingchun Road, Hangzhou, China. E-mail: xmfang@zju.edu.cn

There is no relevant conflict of interest

A running head: Prediction of postoperative infections in elderly

**Appendix 1. Membership of the International Surgical Outcomes Study (ISOS) group in China**

National Co-ordinator: Xiangming Fang

Local investigators:

*local co-ordinator

**Affiliated Hospital of Guiyang Medical College**

Ying Cao, Hong Gao*, Tingju Hu, Jie Lv, Jian Yang, Yang Yang, Yi Zhong, Jing Zhou, Xiaohua Zou

**Affiliated Hospital of Zunyi Medical College**

Miao He, Xiaoying Li, Dihuan Luo, Haiying Wang*, Tian Yu*

**Daping Hospital & Research Institute of Surgery of the Third Military Medical University**

Liyong Chen*, Lijun Wang

**Guangdong Provincial People’s Hospital**

Yunfei Cai, Zhongming Cao, Yanling Li, Jiaxin Lian, Haiyun Sun, Sheng Wang*, Zhipeng Wang, Kenru Wang, Yi Zhu

**Hangzhou Red-cross Hospital**

Xindan Du, Hao Fan, Yunbin Fu, Lixia Huang, Yanming Huang, Haifang Hwan, Hong Luo, Pi-Sheng Qu, Fan Tao*, Zhen Wang, Guoxiang Wang*, Shun Wang, Yan Zhang, Xiaolin Zhang

**Huzhou Central Hospital**

Chao Chen, Weixing Wang*

**Lihuili Hospital, School of Medicine, Ningbo University**

Zhengyuan Liu*

**Lishui People’s Hospital of Zhejiang Province**

Lihua Fan*

**Nanfang Hospital of Southern Medical University**

Jing Tang*

**Ningbo Number 1 Hospital of Zhejiang Province**

Yijun Chen, Yongjie Chen, Yangyang Han, Changshun Huang*, Guojin Liang, Jing Shen, Jun Wang, Qiuhong Yang, Jungang Zhen, Haidong Zhou

**Ningbo Number 2 Hospital of Zhejiang Province**

Junping Chen*, Zhang Chen, Xiaoyu Li, Bo Meng, Haiwang Ye, Xiaoyan Zhang

**Qianfoshan Hospital Affiliated to Shandong University**

Yanbing Bi, Jianqiao Cao, Fengying Guo, Hong Lin, Yang Liu, Meng Lv, Pengcai Shi, Xiumei Song, Chuanyu Sun, Yongtao Sun, Yuelan Wang*, Shenhui Wang, Min Zhang

**Renmin Hospital of Wuhan University**

Rong Chen, Jiabao Hou, Yan Leng, Qing-tao Meng, Li Qian, Zi-ying Shen, Zhong-yuan Xia*, Rui Xue, Yuan Zhang, Bo Zhao, Xian-jin Zhou

**Shanxi Provincial People’s Hospital**

Qiang Chen, Huinan Guo, Yongqing Guo, Yuehong Qi*, Zhi Wang, Jianfeng Wei, Weiwei Zhang, Lina Zheng

**Sir Run Run Shaw Hospital, School of Medicine, Zhejiang University**

Qi Bao, Yaqiu Chen, Yijiao Chen, Yue Fei, Nianqiang Hu, Xuming Hu, Min Lei, Xiaoqin Li, Xiaocui Lv, Jie Lv, Fangfang Miao, Lingling Ouyang, Lu Qian, Conyu Shen, Yu Sun, Yuting Wang, Dong Wang, Chao Wu, Liyuan Xu, Jiaqi Yuan, Lina Zhang, Huan Zhang, Yapping Zhang, Jinning Zhao, Chong Zhao, Lei Zhao, Tianzhao Zheng, Dachun Zhou*, Haiyan Zhou*, Ce Zhou

**Southwest Hospital of Third Military Medical University**

Kaizhi Lu*, Ting Zhao

**Affiliated Hospital of Hangzhou Normal University**

Changlin He*

**First Affiliated Hospital, School of Medicine, Zhejiang University**

Hong Chen, Shasha Chen, Baoli Cheng, Jie He, Lin Jin, Caixia Li, Hui Li, Yuanming Pan, Yugang Shi, Xiao Hong Wen*, Shuijing Wu*, Guohao Xie, Kai Zhang, Bing Zhao

**First Affiliated Hospital of Anhui Medical University**

Xianfu Lu*

**First Affiliated Hospital of Bengbu Medical College**

Feifei Chen, Qisheng Liang*, Xuewu Lin, Yunzhi Ling, Gang Liu, Jing Tao, Lu Yang, Jialong Zhou

**First Affiliated Hospital of Nanchang University**

Fumei Chen, Zhonggui Cheng, Hanying Dai, Yunlin Feng, Benchao Hou, Haixia Gong, Chun hua Hu, Haijin Huang, Jian Huang, Zhangjie Jiang, Mengyuan Li, Jiamei Lin, Mei Liu, Weicheng Liu, Zhen Liu, Zhiyi Liu, Foquan Luo*, Longxian Ma, Jia Min, Xiaoyun Shi, Zhiping Song, Xianwen Wan, Yingfen Xiong, Lin Xu, Shuangjia Yang, Qin Zhang, Hongyan Zhang, Huaigen Zhang, Xuekang Zhang, Lili Zhao, Weihong Zhao, Weilu Zhao, Xiaoping Zhu

**First Affiliated Hospital of Wenzhou Medical University**

Yun Bai, Linbi Chen, Sijia Chen, Qinxue Dai, Wujun Geng, Kunyuan Han, Xin He, Luping Huang, Binbin Ji, Danyun Jia, Shenhui Jin, Qianjun Li, Dongdong Liang, Shan Luo, Lulu Lwang, Yunchang Mo, Yuanyuan Pan, Xinyu Qi, Meizi Qian, Jinling Qin, Yelong Ren, Yiyi Shi, Junlu Wang*, Junkai Wang, Leilei Wang, Junjie Xie, Yixiu Yan, Yurui Yao, Mingxiao Zhang, Jiashi Zhao, Xiuxiu Zhuang

**First Affiliated Hospital of Zhengzhou University**

Yanqiu Ai*, Fang Du, Long He, Ledan Huang, Zhisong Li, Huijuan Li, Yetong Li, Liwei Li, Su Meng, Yazhuo Yuan, Enman Zhang, Jie Zhang, Shuna Zhao

**First University Hospital of China Medical University**

Zhenrong Ji, Ling Pei*, Li Wang

**General Hospital of Tianjin Medical University**

Chen Chen, Beibei Dong, Jing Li, Ziqiang Miao, Hongying Mu, Chao Qin, Lin Su, Zhiting Wen, Keliang Xie*, Yonghao Yu*, Fang Yuan

**The Second Affiliated Hospital of Anhui Medical University**

Xianwen Hu, Ye Zhang*

**The Second Affiliated Hospital of Jiaxing College**

Wangpin Xiao*, Zhipeng Zhu

**The Second Affiliated Hospital of Nanchang University**

Qingqing Dai, Kaiwen Fu, Rong Hu, Xiaolan Hu, Song Huang, Yaqi Li, Yingping Liang, Shuchun Yu*

**The Second Affiliated Hospital of Shanxi Medical University**

Zheng Guo*, Yan Jing, Na Tang, Jie Wu, Dajiang Yuan*, Ruilin Zhang, Xiaoying Zhao

**Shaoxing Hospital of Zhejiang University**

Yuhong Li*

**Third Hospital of Hebei Medical University**

Hui-Ping Bai, Chun-Xiao Liu, Fei-Fei Liu, Wei Ren, Xiu-Li Wang*, Guan-Jie Xu

**Third Xiangya Hospital of Central South University**

Na Hu, Bo Li, Yangwen Ou*, Yongzhong Tang

**Union Hospital, Tongji Medical College, Huazhong University of Science and Technology**

Shanglong Yao*, Shihai Zhang

**Xuan Wu Hospital, Capital Medical University**

Cui-Cui Kong, Bei Liu, Tianlong Wang*, Wei Xiao

**Zhejiang Hospital**

Bo Lu, Yanfei Xia*, Jiali Zhou

**Zhejiang Provincial People’s Hospital**

Fang Cai, Pushan Chen, Shuangfei Hu*, Hongfa Wang, Jie Wu, Qiong Xu

**Zhongda Hospital, Southeast University**

Liu Hu, Liang Jing, Jing Li, Bin Li, Qiang Liu, Yuejiang Liu, Xinjian Lu, Zhen Dan Peng, Xiaodong Qiu, Quan Ren, Youliang Tong, Zhen Wang, Jin Wang, Yazhou Wen, Qiong Wu, Jiangyan Xia, Jue Xie, Xiapei Xiong, Shixia Xu, Tianqin Yang, Hui Ye, Ning Yin*, Jing Yuan, Qiuting Zeng, Baoling Zhang, Kang Zheng

**Zhongshan Hospital Fudan University**

Jing Cang, Shiyu Chen, Fang Du, Yu Fan, Shuying Fu, Xiaodong Ge, Baolei Guo, Wenhui Huang, Linghui Jiang, Xinmei Jiang, Lin Jin, Yi Liu, Yan Pan, Yun Ren, Qi Shan, Jiaxing Wang, Fei Wang, Chi Wu, Xiaoguang Zhang*

**Appendix 2. Detailed definitions of infections**

#### Urinary tract infection

**Definition.** A simplified version of the CDC recommendations defines a urinary tract infection as follows: a positive urine culture of ≥10^5^ colony forming units ml^−1^ with no more than two species of micro-organisms, and with at least one of the following symptoms or signs: fever (>38°C), urgency, frequency, dysuria, suprapubic tenderness, costovertebral angle pain or tenderness with no other recognised cause.^22^

1. **Bloodstream infection**

**Definition.** The CDC defines bloodstream infection as one which meets at least one of the following criteria which should not be related to infection at another site:^22^

1. Patient has a recognised pathogen cultured from one or more blood cultures and the organism cultured from blood is not related to an infection at another site
2. Patient has at least one of the following signs or symptoms: fever >38°C, chills or hypotension, and at least one of the following:
   1. Common skin contaminant cultured from two or more blood cultures drawn on separate occasions
   2. Common skin contaminant cultured from at least one blood culture from a patient with an intravascular line, and the physician institutes appropriate antimicrobial therapy
   3. Positive blood antigen test.

**3. Superficial surgical site infection**

**Definition.** The CDC defines a superficial incisional surgical site infection as one which meets the following criteria.^22^

1. Infection occurs within 30 days after surgery and
2. Involves only skin and subcutaneous tissue of the incision and
3. The patient has at least one of the following:
   1. purulent drainage from the superficial incision
   2. organisms isolated from an aseptically obtained culture of fluid or tissue from the superficial incision
   3. at least one of the following symptoms or signs of infection: pain or tenderness, localised swelling, redness or heat, and superficial incision is deliberately opened by surgeon and is culture positive or not cultured. A culture-negative finding does not meet this criterion.
   4. diagnosis of an incisional surgical site infection by a surgeon or attending physician.

**4. Deep surgical site infection**

**Definition.** The CDC defines a deep incisional surgical site infection as one which meets the following criteria.^22^

1. Infection occurs within 30 days after surgery if no implant is left in place or 1 year if implant is in place.
2. Involves deep soft tissues (e.g. fascial and muscle layers) of the incision.
3. The patient has at least one of the following:
   1. purulent drainage from the deep incision but not from the organ/space component of the surgical site
   2. a deep incision spontaneously dehisces or is deliberately opened by a surgeon and is culture-positive or not cultured when the patient has at least one of the following symptoms or signs: fever (>38°C), or localised pain or tenderness. A culture-negative finding does not meet this criterion.
   3. an abscess or other evidence of infection involving the deep incision is found on direct examination, during surgery, or by histopathological or radiological examination
   4. diagnosis of an incisional surgical site infection by a surgeon or attending physician.

**5. Body cavity infection**

**Definition.** The CDC defines body cavity infection as one which involves any part of the body excluding the fascia, muscle layers, or organs and meets the following criteria.^22^

1. Infection occurs within 30 days after surgery.
2. The infection appears to be related to the surgical procedure and involves any part of the body, excluding the skin incision, fascia or muscle layers opened or manipulated, or organs during the operative procedure.
3. The patient has at least one of the following:
   1. purulent drainage from a drain that is placed through a stab wound into the body cavity
   2. organisms isolated from an aseptically obtained culture of fluid or tissue in the body cavity
   3. an abscess or other evidence of infection involving the body cavity that is found on direct examination, during reoperation or by histopathological or radiological examination
   4. diagnosis of body cavity infection by a surgeon or attending physician.

**6. Pneumonia**

**Definition.** The CDC defines pneumonia as follows:^22^

Two or more serial chest radiographs with at least one of the following (one radiograph is sufficient for patients with no underlying pulmonary or cardiac disease):

1. new or progressive and persistent infiltrates
2. consolidation
3. cavitation;

at least one of the following

1. fever (>38°C) with no other recognised cause
2. leucopaenia (white cell count < 4 × 10^9^ l^−1^) or leucocytosis (white cell count >12 × 10^9^ l^−1^)
3. for adults >70 years old, altered mental status with no other recognised cause;

and at least two of the following

1. new onset of purulent sputum or change in character of sputum, or increased respiratory secretions, or increased suctioning requirements
2. new onset or worsening cough, or dyspnoea, or tachypnoea
3. râles or bronchial breath sounds
4. worsening gas exchange (hypoxaemia, increased oxygen requirement, increased ventilator demand).

**Appendix 3. Logit predictive model**

Pr(PICs=1)= exp(0.0316*(Gender=Male)–0.0127*(ASA=II)+0.0447*(ASA=III)+0.1353*(ASA=IV)+0.0938*(Coronary artery disease=Yes)+0.0268*(Chronic comorbid disease of other=Yes) – 0.0358*(Urology and kidney=Yes) – 0.0383*(Head and neck=Yes)+0.2908*(Cardiac=Yes)-0.0370*(Surgical procedure of other=Yes) – 0.0504*(Sedation/local anesthesia=Yes)-0.0674*(Laparoscopic surgery=Yes) – 0.0271*(Severity of surgical=Intermediate)+0.0800*(Severity of surgery=Major))/(1 + exp(0.0316*(Gender=Male)–0.0127*(ASA=II)+0.0447*(ASA=III)+0.1353*(ASA=IV)+0.0938*(Coronary artery disease=Yes)+0.0268*(Chronic comorbid disease of other=Yes) – 0.0358*(Urology and kidney=Yes) – 0.0383*(Head and neck=Yes)+0.2908*(Cardiac=Yes)-0.0370*(Surgical procedure of other=Yes) – 0.0504*(Sedation/local anesthesia=Yes)-0.0674*(Laparoscopic surgery=Yes) – 0.0271*(Severity of surgical=Intermediate)+0.0800*(Severity of surgery=Major)))

**7 supplemental tables and 6 supplemental figures:**

Tables

Supplemental Table 1. Unadjusted and adjusted odds ratio (OR) and 95% confidence interval (95%CI) using simple logistic regression model and inverse probability weights method

Supplemental Table 2. Some clinical aspects and information related with infections

Supplemental Table 3. Time period for surgery and infection diagnosis according with type of surgery

Supplemental Table 4. Coefficients of logit predictive model using associations of independent risk factors with postoperative infections

Supplemental Table 5: Distributions of independent risk factors among training dataset according to the status of postoperative infections

Supplemental Table 6: Distributions of independent risk factors among validation dataset according to the status of postoperative infections

Supplemental Table 7. Optimal structure of various neural network predictive models

Figures

Supplemental Figure 1. Model for “neural network” analysis process

Supplemental Figure 2. The area under the receivers operating characteristic curve (AUC) for possible structures of neural network predictive model I

Supplemental Figure 3. The optimal structure of the neural network predictive model I

Supplemental Figure 4. The area under the receivers operating characteristic curve (AUC) for possible structures of neural network predictive model II

Supplemental Figure 5. The optimal structure of the neural network predictive model II

Supplemental Table 1. Unadjusted and adjusted odds ratio (OR) and 95% confidence interval (95%CI) using simple logistic regression model and inverse probability weights method

| Risk factors | Unadjusted | | Adjusted | |
| --- | --- | --- | --- | --- |
|  | OR (95% CI) | *P* | OR (95% CI) | *P* |
| Age | 1.02 (1.00,1.05) | 0.036 | 1.00 (1.00,1.00) | 0.220 |
| Gender (Male vs. Female) | 1.57 (1.12,2.20) | 0.008 | 1.03 (1.00,1.06) | 0.023 |
| Current smoker (Yes vs. No) | 1.31 (0.87,1.96) | 0.192 | 1.02 (0.98,1.06) | 0.302 |
| ASA Score |  |  |  |  |
| I |  |  |  |  |
| II | 1.39 (0.73,2.64) | 0.312 | 0.99 (0.96,1.02) | 0.391 |
| III | 3.28 (1.66,6.50) | <0.001 | 1.05 (1.01,1.08) | 0.015 |
| IV | 5.33 (1.52,18.77) | 0.009 | 1.14 (0.95,1.38) | 0.149 |
| Chronic comorbid disease (Yes vs. No) |  |  |  |  |
| Coronary artery disease | 2.80 (1.66,4.74) | <0.001 | 1.110(1.02,1.8) | 0.008 |
| Heart failure | 4.11 (1.31,12.89) | 0.015 | 1.19 (0.96,1.47) | 0.108 |
| Diabetes mellitus | 1.59 (0.98,2.59) | 0.062 | 1.03 (0.99,1.09) | 0.166 |
| Metastatic cancer | 1.59 (0.98,2.59) | 0.062 | 1.05 (0.94,1.18) | 0.389 |
| Cirrhosis | 2.71 (0.58,12.64) | 0.205 | 1.11 (0.88,1.39) | 0.365 |
| Stroke | 0.64 (0.20,2.07) | 0.456 | 0.97 (0.92,1.03) | 0.374 |
| COPD/Asthma | 1.88 (0.83,4.23) | 0.129 | 1.06 (0.97,1.16) | 0.224 |
| Other | 1.33 (0.95,1.86) | 0.091 | 1.03 (1.00,1.05) | 0.048 |
| Blood test results |  |  |  |  |
| Haemoglobin | 0.95 (0.88,1.03) | 0.202 | 1.00 (0.99,1.00) | 0.642 |
| Serum creatinine | 1.11 (0.71,1.72) | 0.655 | 1.01 (0.98,1.03) | 0.695 |
| Sodium | 0.97 (0.93,1.01) | 0.143 | 1.00 (1.00,1.00) | 0.296 |
| Leucocytes | 1.57 (1.03,2.40) | 0.035 | 1.03 (0.99,1.07) | 0.197 |
| Surgical procedure (Yes vs. No) |  |  |  |  |
| Orthopedic | 1.33 (0.89,1.98) | 0.163 | 1.01 (0.98,1.05) | 0.358 |
| Obstetrics and gynaecology | 0.87 (0.35,2.19) | 0.769 | 0.99 (0.93,1.05) | 0.757 |
| Urology and kidney | 0.54 (0.30,0.97) | 0.040 | 0.96 (0.94,0.99) | 0.012 |
| Upper gastrointestinal | 1.91 (0.99,3.68) | 0.053 | 1.06 (0.98,1.14) | 0.121 |
| Lower gastrointestinal | 1.42 (0.83,2.46) | 0.204 | 1.02 (0.97,1.06) | 0.469 |
| Hepatobiliary | 1.17 (0.69,1.99) | 0.554 | 1.00 (0.96,1.04) | 0.899 |
| Vascular | 0.54 (0.13,2.26) | 0.402 | 0.97 (0.91,1.03) | 0.271 |
| Head and neck | 0.50 (0.29,0.86) | 0.012 | 0.96 (0.94,0.99) | 0.005 |
| Cardiac | 7.56 (4.14,13.82) | <0.001 | 1.34 (1.17,1.53) | <0.001 |
| Thoracic | 1.41 (0.74,2.70) | 0.294 | 1.03 (0.97,1.09) | 0.367 |
| Other | 0.55 (0.30,1.01) | 0.054 | 0.96 (0.94,0.99) | 0.010 |
| Anesthetic technique (Yes vs. No) |  |  |  |  |
| General | 1.34 (0.93,1.94) | 0.112 | 1.02 (1.00,1.05) | 0.078 |
| Spinal | 1.16 (0.72,1.88) | 0.548 | 1.00 (0.95,1.05) | 0.931 |
| Epidural | 1.17 (0.75,1.82) | 0.483 | 1.03 (0.98,1.09) | 0.270 |
| Sedation/Local | 0.30 (0.15,0.63) | 0.001 | 0.95 (0.92,0.99) | 0.006 |
| Laparoscopic surgery (Yes vs. No) | 0.33 (0.18,0.62) | <0.001 | 0.94 (0.92,0.97) | <0.001 |
| Cancer surgery (Yes vs. No) | 1.23 (0.84,1.79) | 0.290 | 1.00 (0.97,1.03) | 0.877 |
| Severity of surgical |  |  |  |  |
| Minor |  |  |  |  |
| Intermediate | 1.47 (0.78,2.79) | 0.234 | 0.97 (0.95,0.99) | 0.007 |
| Major | 4.66 (2.58,8.40) | <0.001 | 1.08 (1.06,1.11) | <0.001 |
| Surgical check list used (Yes vs. No) | 1.76 (0.55,5.69) | 0.341 | 1.03 (0.98,1.09) | 0.198 |

ASA: American Society of Anesthesiologists; COPD: Chronic obstructive pulmonary disease

Supplemental Table 2. Some clinical aspects and information related with infections

|  | Overall | No Postoperative infections | Postoperative infections | *P* |
| --- | --- | --- | --- | --- |
| Medication (Yes, %)  Nutrition Support (Yes, %)  Time period from baseline evaluation and surgery (Days, SD) | 198 (9.8)  427 (21.2)  2.11 (0.50) | 177 (9.5)  400 (21.5)  2.11 (0.49) | 21 (13.6)  27 (17.5)  2.12 (0.56) | 0.131  0.291  0.793 |
| Surgical procedure category (%)  Breast  Cardiac  Head and neck  Hepato-biliary  Lower gastro-intestinal  Gynaecology  Orthopaedic  Other  Plastics/Cutaneous  Thoracic (gut)  Thoracic (lung & other)  Upper gastro-intestinal  Urology & Kidney  Vascular  Days in hospital after surgery (SD) | 41(2.0)  50 (2.5)  347 (17.2)  195 (9.7)  156 (7.7)  74 (3.7)  361 (17.9)  259 (12.9)  12 (0.6)  36 (1.8)  71 (3.5)  83 (4.1)  283 (14.1)  46 (2.3)  8.21 (5.82) | 41 (2.2)  32 (1.7)  332 (17.8)  178 (9.6)  140 (7.5)  69 (3.7)  327 (17.6)  247 (13.3)  12 (0.6)  32 (1.7)  64 (3.4)  72 (3.9)  270 (14.5)  44 (2.4)  7.89 (5.61) | 0 (0.0)  18 (11.7)  15 (9.7)  17 (11.0)  16 (10.4)  5 (3.2)  34 (22.1)  12 (7.8)  0 (0.0)  4 (2.6)  7 (4.5)  11 (7.1)  13 (8.4)  2 (1.3)  12.06 (6.82) | <0.001  <0.001 |

Supplemental Table 3. Time period for surgery and infection diagnosis according with type of surgery

|  | Time period for surgery and  infection diagnosis (Days, SD) P | | | |  |
| --- | --- | --- | --- | --- | --- |
| Type of surgery | |  | 0.851 | | |
| Overall | | 2.21 (0.81) |  | | |
| Cardiac | | 2.17 (0.86) |  | | |
| Head and neck | | 2.53 (1.30) |  | | |
| Hepato-biliary | | 2.00 (0.35) |  | | |
| Lower gastro-intestinal | | 2.31 (0.95) |  | | |
| Gynaecology | | 2.20 (1.10) |  | | |
| Orthopaedic | | 2.21 (0.91) |  | | |
| Other | | 2.00 (0.60) |  | | |
| Thoracic (gut) | | 2.00 (0.00) |  | | |
| Thoracic (lung&other) | | 2.57 (0.79) |  | | |
| Upper gastro-intestinal | | 2.18 (0.40) |  | | |
| Urology&Kidney | | 2.15 (0.55) |  | | |
| Vascular | | 2.00 (0.00) | |  | |

Supplemental Table 4. Coefficients of logit predictive model using associations of independent risk factors with postoperative infections

| Risk factors | Coefficients |
| --- | --- |
| Gender (Male) | 0.0317 |
| ASA Score |  |
| II | -0.0127 |
| III | 0.0447 |
| IV | 0.1353 |
| Chronic comorbid disease (Yes) |  |
| Coronary artery disease | 0.0938 |
| Other | 0.0268 |
| Surgical procedure (Yes) |  |
| Urology and kidney | -0.0358 |
| Head and neck | -0.0383 |
| Cardiac | 0.2908 |
| Other | -0.0370 |
| Sedation/Local anesthesia (Yes) | -0.0504 |
| Laparoscopic surgery (Yes) | -0.0574 |
| Severity of surgery |  |
| Intermediate | -0.0271 |
| Major | 0.0800 |

ASA: American Society of Anesthesiologists

Supplemental Table 5 Distributions of independent risk factors among training dataset according to the status of postoperative infections

| Independent risk factors | Overall | No Postoperative infections | Postoperative infections | *P* |
| --- | --- | --- | --- | --- |
| n | 1510 | 1394 | 116 |  |
| Gender (Male, %) | 754 (49.9) | 682 (48.9) | 72 (62.1) | 0.009 |
| ASA score (%) |  |  |  | < 0.001 |
| I | 172 (11.4) | 163 (11.7) | 9 (7.8) |  |
| II | 1077 (71.3) | 1006 (72.2) | 71 (61.2) |  |
| III | 247 (16.4) | 214 (15.4) | 33 (28.4) |  |
| IV | 14 ( 0.9) | 11 ( 0.8) | 3 ( 2.6) |  |
| Chronic comorbid disease (Yes, %) |  |  |  |  |
| Coronary artery disease | 74 ( 4.9) | 62 ( 4.4) | 12 (10.3) | 0.009 |
| Other | 546 (36.2) | 495 (35.5) | 51 (44.0) | 0.085 |
| Surgical procedure (Yes, %) |  |  |  |  |
| Urology and kidney | 211 (14.0) | 201 (14.4) | 10 ( 8.6) | 0.112 |
| Head and neck | 252 (16.7) | 241 (17.3) | 11 ( 9.5) | 0.042 |
| Cardiac | 38 ( 2.5) | 24 ( 1.7) | 14 (12.1) | <0.001 |
| Other | 200 (13.2) | 191 (13.7) | 9 ( 7.8) | 0.095 |
| Sedation/Local anesthesia (Yes, %) | 229 (15.2) | 224 (16.1) | 5 ( 4.3) | 0.001 |
| Laparoscopic surgery (Yes, %) | 264 (17.5) | 258 (18.5) | 6 ( 5.2) | <0.001 |
| Severity of surgical (%) |  |  |  |  |
| Minor | 301 (19.9) | 289 (20.7) | 12 (10.3) | <0.001 |
| Intermediate | 635 (42.1) | 604 (43.3) | 31 (26.7) |  |
| Major | 574 (38.0) | 501 (35.9) | 73 (62.9) |  |

ASA: American Society of Anesthesiologists; PICs: Postoperative infectious complications

Supplemental Table 6. Distributions of independent risk factors among validation dataset according to the status of postoperative infections

| Independent risk factors | Overall | No Postoperative infections | Postoperative infections | *P* |
| --- | --- | --- | --- | --- |
| n | 504 | 466 | 38 |  |
| Gender (Male, %) | 255 (50.6) | 234 (50.2) | 21 (55.3) | 0.667 |
| ASA score (%) |  |  |  | 0.007 |
| I | 59 (11.7) | 57 (12.2) | 2 (5.3) |  |
| II | 368 (73.0) | 345 (74.0) | 23 (60.5) |  |
| III | 72 (14.3) | 60 (12.9) | 12 (31.6) |  |
| IV | 5 ( 1.0) | 4 ( 0.9) | 1 ( 2.6) |  |
| Chronic comorbid disease (Yes, %) |  |  |  |  |
| Coronary artery disease | 34 ( 6.7) | 27 ( 5.8) | 7 (18.4) | 0.008 |
| None of the below | 244 (48.4) | 230 (49.4) | 14 (36.8) | 0.188 |
| Other | 190 (37.7) | 175 (37.6) | 15 (39.5) | 0.952 |
| Surgical procedure (%) |  |  |  |  |
| Urology and kidney | 72 (14.3) | 69 (14.8) | 3 ( 7.9) | 0.352 |
| Head and neck | 95 (18.8) | 91 (19.5) | 4 (10.5) | 0.251 |
| Cardiac | 12 ( 2.4) | 8 ( 1.7) | 4 (10.5) | 0.004 |
| Other | 59 (11.7) | 56 (12.0) | 3 ( 7.9) | 0.619 |
| Sedation/Local anesthesia (%) | 63 (12.5) | 60 (12.9) | 3 ( 7.9) | 0.524 |
| Laparoscopic surgery (%) | 96 (19.0) | 91 (19.5) | 5 (13.2) | 0.455 |
| Severity of surgical (%) |  |  |  | <0.001 |
| Minor | 106 (21.0) | 105 (22.5) | 1 ( 2.6) |  |
| Intermediate | 206 (40.9) | 198 (42.5) | 8 (21.1) |  |
| Major | 192 (38.1) | 163 (35.0) | 29 (76.3) |  |

ASA: American Society of Anesthesiologists

Supplemental Table 7. Optimal structure of various neural network predictive models

|  | Layer | Neuron1 | Neuron2 | AUC Valid | Model | seed |
| --- | --- | --- | --- | --- | --- | --- |
| Model I | 1 | 11 | 3 | 0.6407556 | 11-3-1 | 48 |
| Model II | 1 | 28 | 24 | 0.7625367 | 28-24-1 | 375 |

AUC: The area under the receivers operating characteristic curve

Supplemental Figure 1. Model for “neural network” analysis process

Supplemental Figure 2. The area under the receivers operating characteristic curve (AUC) for possible structures of neural network predictive model I

Supplemental Figure 3. The optimal structure of the neural network predictive model I.

Supplemental Figure 4. The area under the receivers operating characteristic curve (AUC) for possible structures of neural network predictive model II

Supplemental Figure 5. The optimal structure of the neural network predictive model II
